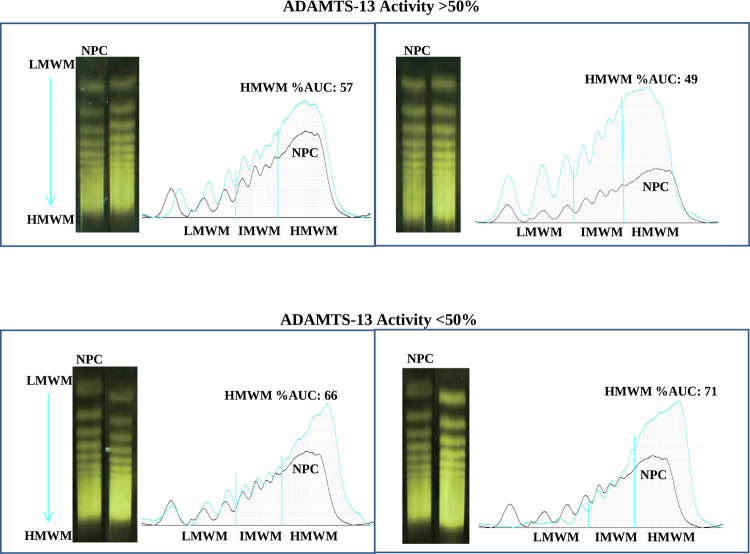# A comparative study of anti–ADAMTS-13 antibody dynamics in immune-mediated thrombotic thrombocytopenic purpura

**DOI:** 10.1016/j.rpth.2024.102525

**Published:** 2024-07-22

**Authors:** Maria Rita Cozzi, Fabio Del Ben, Chiara Corso, Agostino Steffan

**Affiliations:** Centro di Riferimento Oncologico (CRO) Aviano, National Cancer Institute, Istituto di Ricovero e Cura a Carattere Scientifico (IRCCS), Immunopathology and Cancer Biomarkers Unit, Department of Cancer Research and Advanced Diagnostics, Aviano, Italy

**Keywords:** acquired ADAMTS13 protein, ADAMTS-13, anti–ADAMTS-13 autoantibodies, enzyme-linked immunosorbent assay, immune-mediated thrombotic thrombocytopenic purpura, luminescent measurements, purpura, thrombotic thrombocytopenic, thrombotic thrombocytopenic purpura, von Willebrand factor

## Abstract

**Background:**

Thrombotic thrombocytopenic purpura, particularly its immune-mediated variant (iTTP), necessitates accurate diagnostic approaches for effective management.

**Objectives:**

To compare a chemiluminescence immunoassay (CLIA) and an enzyme-linked immunosorbent assay (ELISA) for testing ADAMTS-13 activity and detecting anti–ADAMTS-13 autoantibodies (AAbs) in patients with iTTP.

**Methods:**

This study involved 31 paired samples from 12 iTTP patients. ADAMTS-13 activity was measured using the HemosIL AcuStar (Instrumentation Laboratory, CLIA) and Technozym (Technoclone) activity assay (ELISA). The presence of AAbs was assessed using Technozym ADAMTS-13-INH assay (ELISA) and HemosIL AcuStar activity (CLIA) within a Bethesda assay following mixing with normal pool plasma. von Willebrand factor (VWF) multimers were analyzed using the HYDRASYS-2 SCAN system and the HYDRAGEL 5- or 11-VW Multimer kits (Sebia). VWF activity levels were measured with the HemosIL AcuStar VWF:GPIbR on the ACL AcuStar Analyzer (IL).

**Results:**

For ADAMTS-13 activity, a strong linear relationship and no bias between CLIA and ELISA were confirmed (slope = 1.01 [0.91, 1.11], intercept = 0.00 [−0.47, 0]). However, significant discrepancies were found in AAb detection during remission phases with ADAMTS-13 activity between 10% and 50%, with CLIA and ELISA showing significant divergence (*P* < .001, Cohen’s *g* = 0.34). Consistently, VWF multimers and activity levels exhibited significantly different values between remission samples with ADAMTS-13 activity below 50% and above 50%. In longitudinal analysis of patients with multiple iTTP relapses, positivity to CLIA appears to precede ELISA in predicting exacerbations.

**Conclusion:**

While CLIA and ELISA might be interchangeable for assessing ADAMTS-13 activity, they are not equivalent for detecting AAbs, particularly in patients in clinical remission with ADAMTS-13 activity between 10% and 50%.

## Essentials

• Immune-mediated thrombotic thrombocytopenic purpura (iTTP) requires accurate diagnostics.
• This study compared chemiluminescence-based immunoassay and enzyme-linked immunosorbent assay for ADAMTS-13 activity and anti–ADAMTS-13 antibody in immune-mediated thrombotic thrombocytopenic purpura.
• ADAMTS-13 activity measurement was similar by both methods; anti–ADAMTS-13 autoantibody detection differed significantly.
• The difference is focused in patients in clinical remission with ADAMTS-13 activity between 10% and 50%.

## 1 Introduction

Thrombotic thrombocytopenic purpura (TTP), also known as Moschcowitz syndrome, is an uncommon hematological disorder caused by deficiency in ADAMTS-13, a von Willebrand factor (VWF) cleaving protein. TTP presents in both inherited and acquired forms. The inherited form, often referred to as congenital TTP, is rare and typically manifests in early childhood. The acquired form, known as immune-mediated TTP (iTTP), is more prevalent and accounts for approximately 95% of all TTP cases. iTTP is typically acute, progressing rapidly over a few days, so achieving a timely diagnosis is absolutely crucial. Unfortunately, iTTP remains a condition with a clinical presentation that may be mistaken for other disorders, such as hemolytic uremic syndrome, autoimmune diseases, and complications related to pregnancy. Additionally, throughout life, episodes of iTTP may occur as single or repeated events; therefore, it will be essential to perform clinical and laboratory monitoring of these patients over time.

In iTTP, autoantibodies inhibit the activity of ADAMTS-13 and can also remove it from the circulatory system.

The primary function of ADAMTS-13 is to cleave large multimers of VWF, critical in the blood clotting process, by facilitating platelet adhesion and aggregation at sites of blood vessel injury [1]. When ADAMTS-13 is lacking, these VWF multimers with exceptionally high molecular weight (referred to as unusually large-molecular-weight multimers) accumulate on the inner lining of blood vessels or injury sites and form platelet-rich thrombi.

Conditions of high shear, such as those found in the microcirculation of organs like the brain, heart, and kidneys, contribute to the development of platelet-rich thrombi, causing varying degrees of organ damage [2].

The activity of ADAMTS-13, presence of anti–ADAMTS-13 autoantibodies (AAbs), multimeric profile, and VWF activity can be probed through specialistic laboratory assays highlighting different aspects of iTTP [3].

A crucial laboratory test to differentiate iTTP from other thrombotic microangiopathic hemolytic anemias involves assessing the ADAMTS-13 activity and AAb levels in the patient’s plasma. Detecting these antibodies is crucial for distinguishing between congenital and acquired forms of the disease and guiding subsequent treatment decisions. Moreover, AAb dynamics might help understand pathophysiological mechanism of the disease. ADAMTS-13 activity monitoring is used in evaluating the response to therapy and assessing the risk of recurrence.

The latest International Society on Thrombosis and Haemostasis guidelines for diagnosing iTTP underscore the significance of ADAMTS-13 testing (both activity and AAb levels) in conjunction with the pretest probability determined through clinical evaluation when initiating treatment with plasma exchange and corticosteroids for acute patients [4].

Severe deficiency of ADAMTS-13 activity (<10 IU/dL or <10% of normal values) and the presence of AAbs are distinctive and well-established biomarkers for diagnosing iTTP in the acute phase. These patients are generally treated with caplacizumab and rituximab [5].

In long-term clinical remission patients, ADAMTS-13 activity levels may fluctuate, indicating the potential presence of subclinical disease and the risk of clinical relapse. In such cases, when ADAMTS-13 activity levels are below the lower limit of normal, antibody titers are typically undetectable and do not always correlate with the occurrence of clinical relapse. Consequently, monitoring and managing these patients is less well-established and requires frequent serial testing over time.

Hence, the accuracy, accessibility, and turnaround time of plasma ADAMTS-13 activity and AAb tests can significantly influence the diagnosis and the approach to iTTP patients.

Currently, various commercial kits are accessible for routine laboratory use [6]; however, they often involve time-consuming procedures with analytical times ranging from around 30 minutes to several hours and involve complex technical requirements. Moreover, the assessment of ADAMTS-13 AAb through the activity assay, in conjunction with mixture studies, has not undergone extensive investigation compared with the performance of immunologic assays for detecting ADAMTS-13 autoantibodies.

The commonly used tests to measure ADAMTS-13 activity include enzyme-linked immunosorbent assay (ELISA), fluorescence resonance energy transfer, and quantitative chemiluminescence-based immunoassay (CLIA). Numerous studies have demonstrated the suitability of these methods for diagnosing TTP, highlighting their good analytical sensitivity [[7], [8], [9]].

In recent years, there has been a rise in the interest in physiopathologic mechanisms of iTTP, given the clinical impact that they may have with the discovery of new targeted therapeutics, and there is ongoing debate regarding whether the presence of ADAMTS-13 autoantibodies can reliably predict disease recurrence [10]. This growing interest is reflected in a consistent rise in requests for ADAMTS-13 tests referred from hematologists linked to advancements in diagnosing and managing acute TTP patients during follow-up. Therefore, we started to replace the ELISA with the automated CLIA, which has been extensively correlated [11].

In this context, after a preliminary comparison of ADAMTS-13 activity, we explored functional AAb detection via mixing studies, comparing results with conventional immunologic assays.

During remission phases, high-molecular-weight multimers (HMWMs) and VWF activity testing complemented ADAMTS-13 assessment, facilitating comprehensive follow-up and potentially early detection of subclinical iTTP cases.

Finally, we conducted a longitudinal investigation of laboratory findings in patients with multiple relapses to explore the correlation between ADAMTS-13 activity, functional AAb, and iTTP relapse dynamics.

## 2 Methods

### 2.1 Patient samples

Residual plasma samples from 12 patients (4 females with a median age of 69 years, ranging from 35 to 73 years, and 8 males with a median age of 51 years, ranging from 31 to 81 years) submitted for clinical testing were utilized in the study. This study was conducted in accordance with Italian regulations and guidelines. Analyses were performed on routine laboratory tests collected for clinical purposes, with no additional blood draws or examinations conducted outside laboratory activities indicated in the medical prescriptions. Consequently, informed consent and institutional review board approval are embedded in the clinical practice. The double testing using ELISA and CLIA was part of an internal procedure during the laboratory's transition from ELISA to CLIA. Retrospective analysis for research purposes of data collected for clinical purposes is permissible without informed consent in IRCCS (Istituti di Ricovero e Cura a Carattere Scientifico) in Italy, as per the guidelines set forth by the Italian Data Protection Authority, based on Article 110-bis, paragraph 4 of Legislative Decree 196/2003. All data were fully anonymized to ensure patient privacy. All samples, which were collected between 2018 and 2022, had been referred for ADAMTS-13 evaluation after clinical diagnosis or relapse of acquired iTTP and during monitoring in the follow-up period. Peripheral venous blood was obtained using tubes containing citrate anticoagulant solution (sodium citrate) at a final concentration of 0.106 mol/L (Sarstedt), and within 2 hours of collection, it was subjected to centrifugation at 2000 *g* for 15 minutes. The resulting plasma was then frozen at –80 °C until the time of analysis. Patients with acute iTTP were diagnosed based on clinical and laboratory tests according to the criteria outlined in guidelines [4,12]. They exhibited severe thrombocytopenia, microangiopathic hemolytic anemia with varying degrees of organ damage, and plasma ADAMTS-13 activity levels below 10% upon admission. Additionally, plasma samples were collected during clinical remission in the follow-up period. Samples from 20 healthy subjects aged 18 to 65 years with no history of TTP were selected as control materials for measuring ADAMTS-13 activity, AAb levels, VWF activity, and HMWM.

### 2.2 Laboratory tests for ADAMTS-13 activity

A total of 31 distinct residual samples were utilized to assess ADAMTS-13 activity through the HemosIL AcuStar ADAMTS-13 Activity Assay (Instrumentation Laboratory) following the manufacturer’s instructions. This assay employs chemiluminescence technology and has an analytical time of 30 minutes for the quantitative measurement of ADAMTS-13 activity on the ACL AcuStar Analyzer [7].

ADAMTS-13 activity levels were simultaneously analyzed using the Technozym ADAMTS-13 Activity Assay (Technoclone GmbH; ELISA). The analytical time for quantitatively measuring ADAMTS-13 activity is 3 hours using the Tecan Infinite F200 ELISA reader with Magellan software (Tecan Trading AG).

### 2.3 Laboratory tests for anti–ADAMTS-13 antibodies

We utilized the Technozym ADAMTS-13-INH ELISA (Technoclone), as previously described [13], to detect antibodies against ADAMTS-13 following the manufacturer’s guidelines. A comparative experiment for AAb titers was conducted using the HemosIL AcuStar ADAMTS-13 Activity Assay (Instrumentation Laboratory; CLIA). The HemosIL AcuStar ADAMTS-13 Activity Assay can be employed to assess AAb following the preanalytical mixing of the sample with normal pool plasma in a 1:1 ratio (Bethesda assay) [7,14]. In brief, patient and control plasma were heated at 56 °C for 1 hour, and then, dilutions were incubated with untreated pooled normal plasma for 30 minutes at 37 °C. After incubation, the residual ADAMTS-13 activity in the patient and control mixtures was measured using the HemosIL AcuStar ADAMTS-13 Activity Assay. If an immunoglobulin (Ig) G inhibitor is present in the patient sample, the ADAMTS-13 activity in the test sample will be lower than that in the control. The residual ADAMTS-13 activity level (% RA) was calculated using the following formula:

% activity patient mix / % activity control mix, with the residual activity being higher than 75% in all control mixtures.

Accurate calculation of Bethesda units (BUs) requires a range of residual activity of 25% to 75%, while for patients with residual activity below 25%, accurate quantification is not possible without serial dilutions. This would involve substantial reagent consumption and time-consuming operations. Given the focus of our study, this approach would have led to unnecessary use of resources without providing additional insight into our primary objective. In contrast, indicating a positive/negative test offers a more efficient and equally reliable means of identifying inhibitors, which we deemed sufficient for the purposes of this study. For these reasons, we did not perform BU quantification.

### 2.4 von Willebrand activity and multimer assay

For the evaluation of VWF activity, we utilized an automated latex-enhanced immunoassay (HemosIL AcuStar VWF:GPIbR) performed on the ACL AcuStar Analyzer from Instrumentation Laboratory. The assay utilizes magnetic particles coated with a recombinant fragment of platelet GPIb along with the aggregating agent ristocetin [15].

The von Willebrand multimer assay was performed using the HYDRASYS-2 SCAN system and the HYDRAGEL 5- or 11-VW Multimer kits (H5/11VWM, Sebia). Visualization was achieved with the Phoresis software, version 8.63 (Sebia) [16]. Multimer fractions were categorized as follows: low-molecular-weight multimers encompassed peaks 1 to 3, intermediate-molecular-weight multimers) included peaks 4 to 7, and HMWMs consisted of peaks above 7. The area under the curve (AUC) for each fraction was automatically calculated and reported as relative AUC. To ensure accuracy, a normal plasma control (NPC) was included in each gel run.

### 2.5 Statistical analysis

Statistical analysis and data visualization were performed in R version v4.3.2 (October 31, 2023) [17]. Main packages used were tidyverse v2.0.0 [18], ggstatsplot v0.12.2 [19], effectsize v0.8.6 [20], ggplot2 v3.4.4 [21], and mcr v1.3.3 [22].

**Figure 1:**
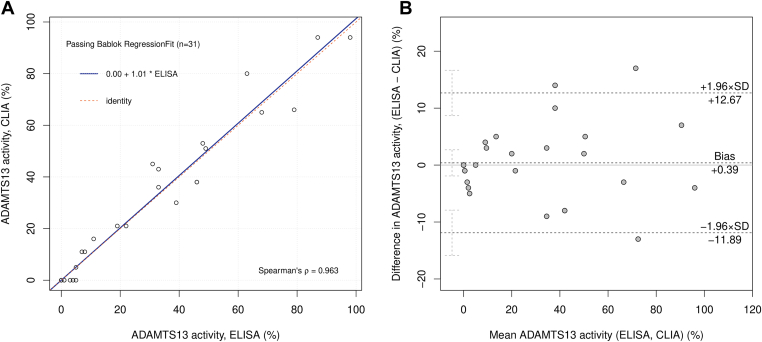
(A) The Passing–Bablok regression analysis comparing the AcuStar (CLIA) and Technozym (ELISA) assays is depicted. The regression line, denoted in blue, is accompanied by a 95% CI. The identity line is illustrated in red. The equation of the regression line is displayed in the top-left corner, while the Spearman correlation coefiicient is presented in the bottom-right corner. (B) The Bland–Altman graph, also known as a mean difference plot, is provided. On the x-axis, the mean value between each pair of measurements is depicted, while the y-axis represents the difference between each pair of measurements. Horizontal dotted lines indicate the mean difference ± 1.96 SDs. CLIA, chemiluminescence immunoassay; ELISA, enzyme-linked immunosorbent assay.

**Figure 2:**
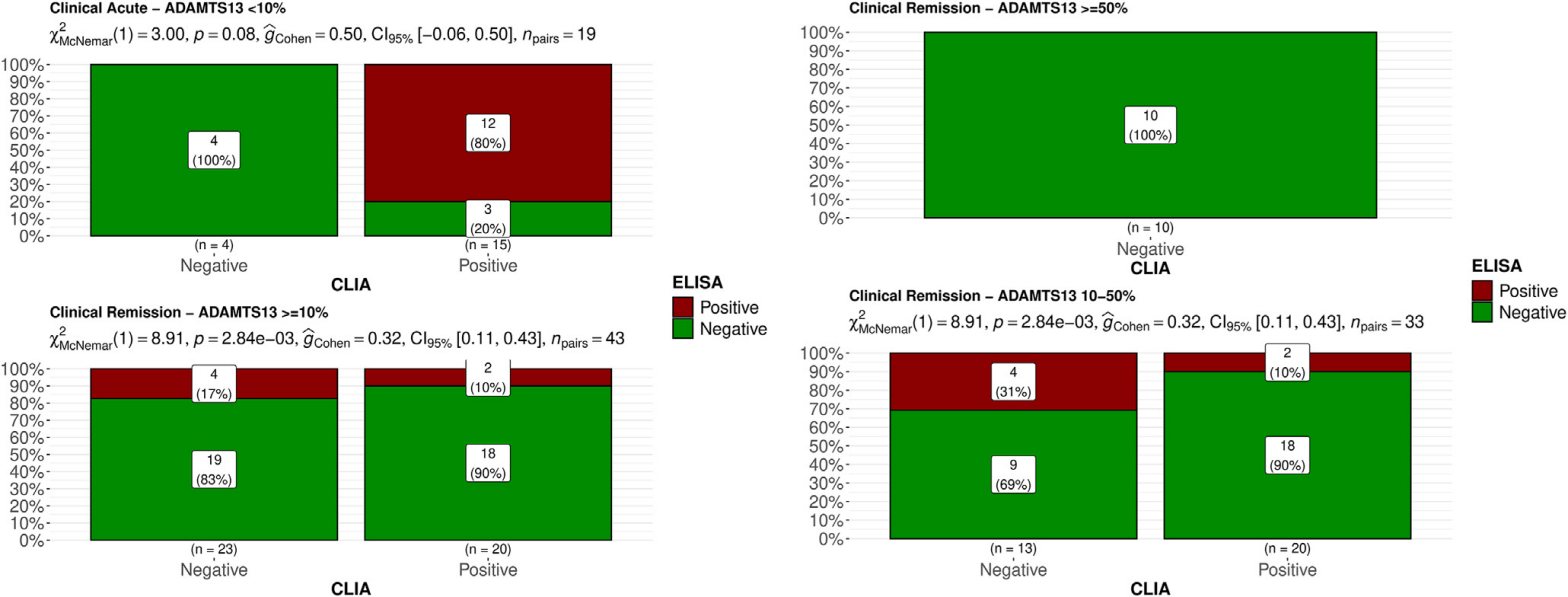
On the left panel, the presence of AAb, identified through ELISA or CLIA, is shown in patients grouped by clinical condition: clinical acute (top-left) or clinical remission (bottom-left). On the right panel, the presence of AAbs, identified through ELISA or CLIA, is shown in patients in clinical remission grouped by ADAMTS-13 activity: ≥50% (top-right) or 10% to 50% (bottom-right). On the x-axis, results from CLIA are represented, while on the y-axis, the percentage of samples tested with ELISA within each CLIA group is depicted. Bars are color-coded according to the results from ELISA. Results from the McNemar test and effect size (Cohen’s *g*) are annotated above the figure. AAb, anti–ADAMTS-13 autoantibody; CLIA, chemiluminescence immunoassay; ELISA, enzyme-linked immunosorbent assay.

**Figure 3:**
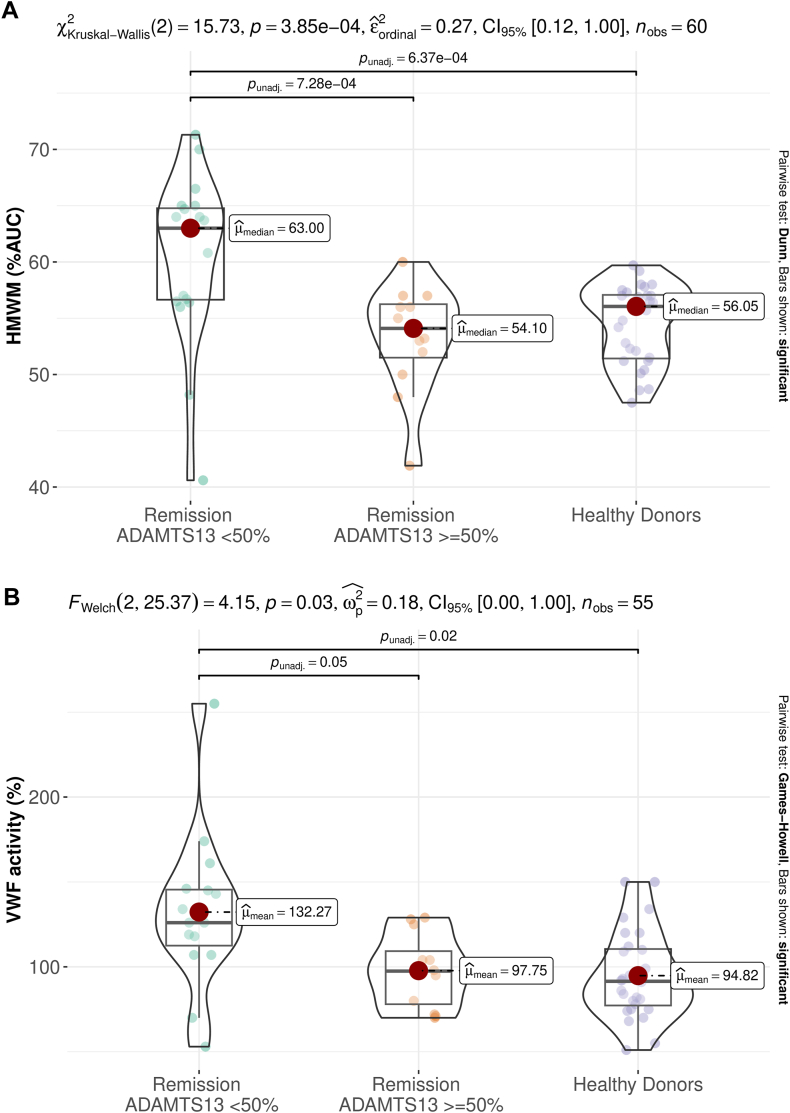
(A) HMWM and (B) VWF activity in samples with iTTP in remission and ADAMTS-13 activity below 50%, above or equal to 50%, and healthy donors. Results from statistical tests are annotated in the figure. HMWM, high-molecular-weight multimers; iTTP, Immune-mediated thrombotic thrombocytopenic purpura; VWF, von Willebrand factor.

**Figure 4:**
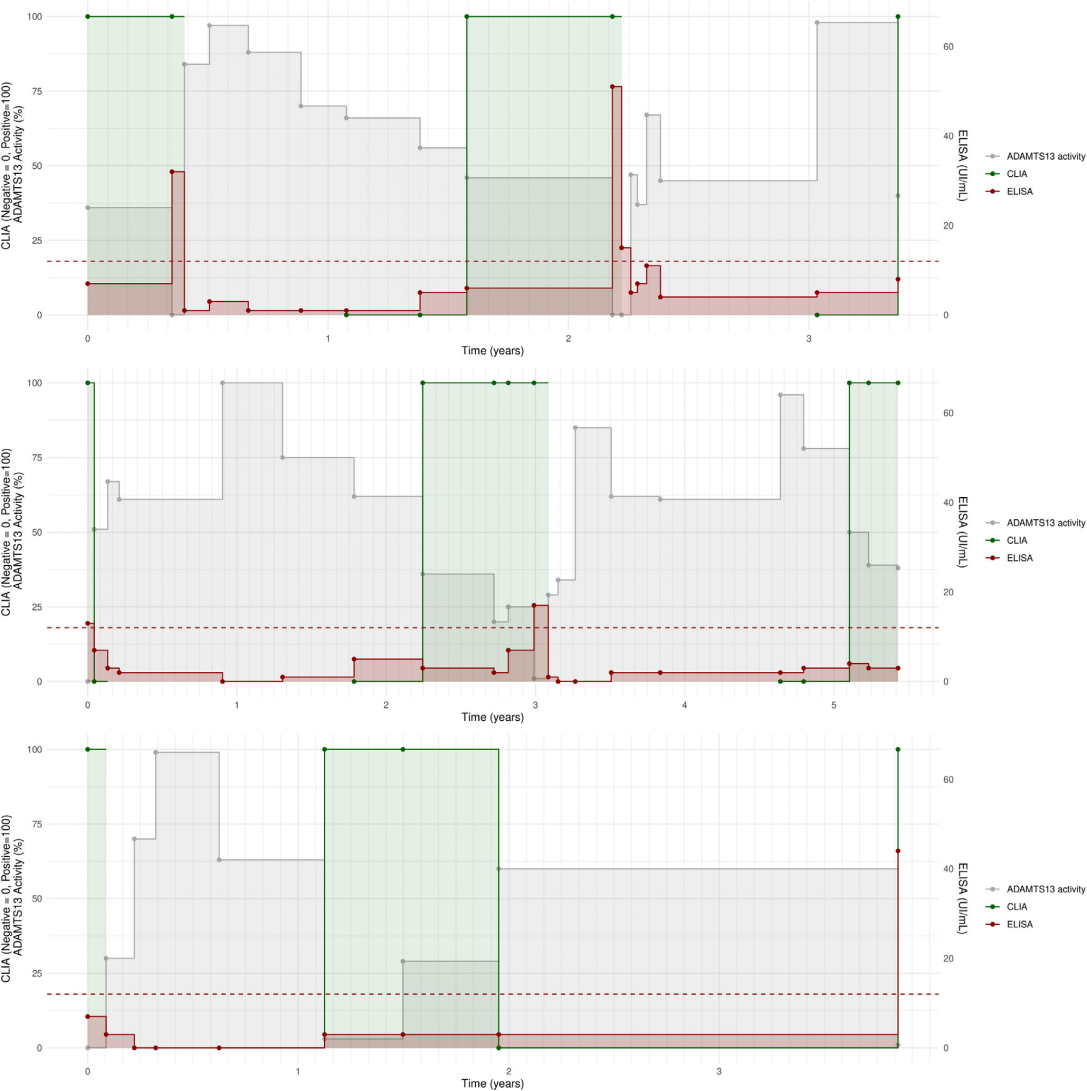
ADAMTS-13 activity (gray), AAb by CLIA (green), and AAb by ELISA (red) over time in single patients. All panels show how the AAb becomes detectable by CLIA as soon as ADAMTS-13 activity falls below 50%, while ELISA starts detecting the AAb later on, when activity further decreases. In the top panel, ELISA is well above the threshold; in the middle panel, it is close to the threshold; and in the bottom panel, it failed to detect the presence of the AAb. The green line is discontinuous because the samples were tested for AAb with CLIA only when ADAMTS-13 activity fell below 75% and until ELISA result was positive. AAb, anti–ADAMTS-13 autoantibody; CLIA, chemiluminescence immunoassay; ELISA, enzyme-linked immunosorbent assay.

Regression was performed according to the Passing–Bablok method. To decide between parametric or nonparametric approaches, Shapiro–Wilk normality test was performed. Parametric hypothesis testing on paired samples (ADAMTS-13 activity) was performed using paired Student’s *t*-test, and effect size was measured with Hedges’ *g*. Hypothesis testing on categorical variables (ADAMTS-13 activity and AAb) on paired samples was performed with McNemar test, and effect size was measured with Cohen’s *g*. Interassay agreement was measured in percentage of concordant result and with Cohen’s *k*. Nonparametric unpaired comparison between groups (HMWM) was performed with Kruskal–Wallis’ 1-way analysis of variance, and effect size was measured with rank epsilon-squared. Similarly, parametric unpaired comparison between groups (VWF) was performed with Welch’s 1-way analysis of variance, and effect size was measured with partial omega-squared. Pairwise comparison of the parametric multiple group comparison was performed with Games–Howell test, which does not require additional *P* adjustment. Pairwise comparison of the nonparametric multiple group comparison was performed with Dunn test, and *P* values were adjusted with the Holm method. In Figure 1, Figure 2, Figure 3, Figure 4, the names of the employed statistical test, results, and CIs are annotated; where available, Bayesian statistics was also annotated in the figures.

## 3 Results

### 3.1 ADAMTS-13 activity

**Supplementary Figure S1:**
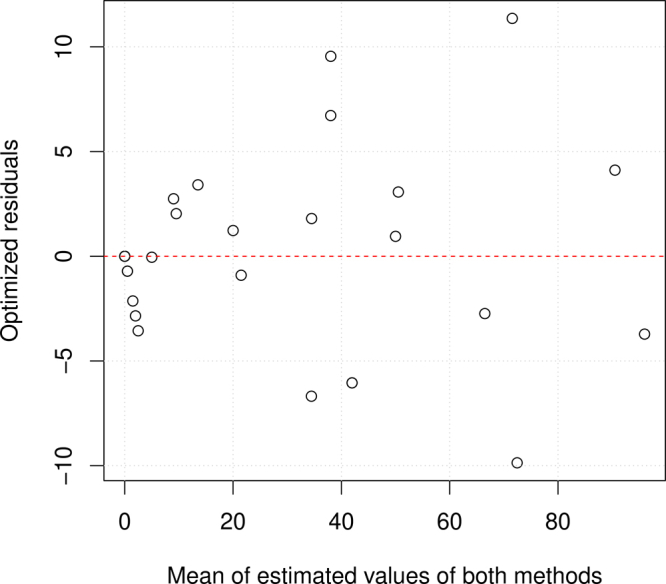


**Supplementary Figure S2:**
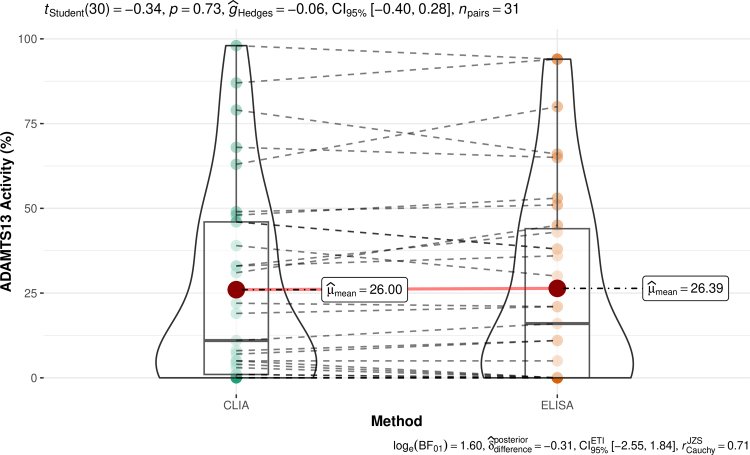


**Supplementary Figure S3:**
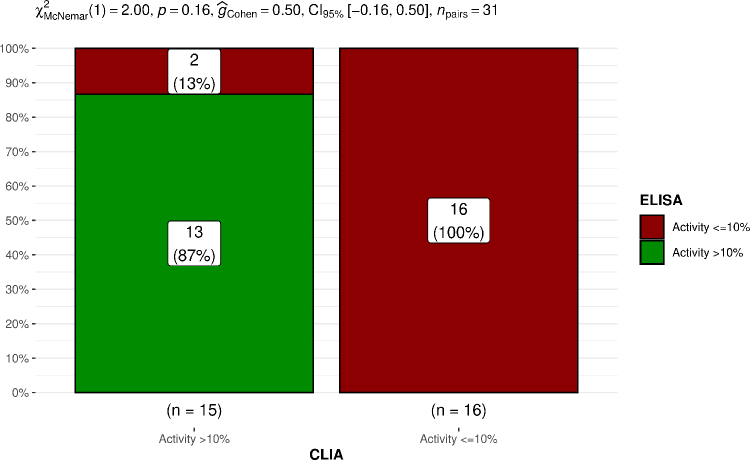


As a preliminary investigation, we compared results of 2 commercially available kits for evaluating ADAMTS-13 activity: the HemosIL AcuStar ADAMTS-13 Activity Assay (CLIA) and the Technozym ADAMTS-13 Activity Assay (ELISA). The evaluation was conducted using a panel of 31 paired samples from 12 patients, encompassing the entire spectrum of values (range <10%-98%). The Spearman’s coefiicient of correlation was 0.96, indicating a very strong correlation, as expected in a method comparison. Passing–Bablok regression analysis, as illustrated in Figure 1A, yielded a regression line closely approximating the identity line, with a slope of 1.01 (0.91, 1.11) and an intercept of 0.00 (−0.47, 0.00). Residuals as shown in the residual plot (Supplementary Figure S1) were randomly scattered around the horizontal line, indicating the absence of proportional bias. Bland–Altman analysis revealed the absence of significant bias, with the mean difference overlapping 0 (0.39 [−1.91 to −2.68]), lower limit of agreement of −11.89 (−15.87 to −7.91), and upper limit of 12.67 (8.69 to 16.65). In paired hypothesis testing, no significant difference was observed between CLIA and ELISA (median, 26.0% vs 26.4%; *P* = .73), as depicted in Supplementary Figure S2. From a diagnostic standpoint, an ADAMTS-13 activity test is considered positive when <10%; otherwise, it is considered negative. Based on this classification, no significant difference was observed between CLIA and ELISA (*P* = .16), as shown in Supplementary Figure S3, while an almost perfect agreement (93.5%, Cohen’s *k* = 0.87) was noted.

### 3.2 Anti–ADAMTS-13 antibodies

We conducted a comparative analysis between 2 assays to evaluate AAbs. The tests under comparison were HemosIL AcuStar ADAMTS-13 Activity Assay (Functional, CLIA) and the Technozym ADAMTS-13-INH assay (Immunologic, ELISA).

An initial assessment utilized 31 samples obtained from 12 patients diagnosed with iTTP, either in the acute or remission phase, tested in parallel with ELISA or CLIA. ELISA positivity was defined as >12 UI/mL, while CLIA positivity was defined as residual activity of <75%.

**Supplementary Figure S4:**
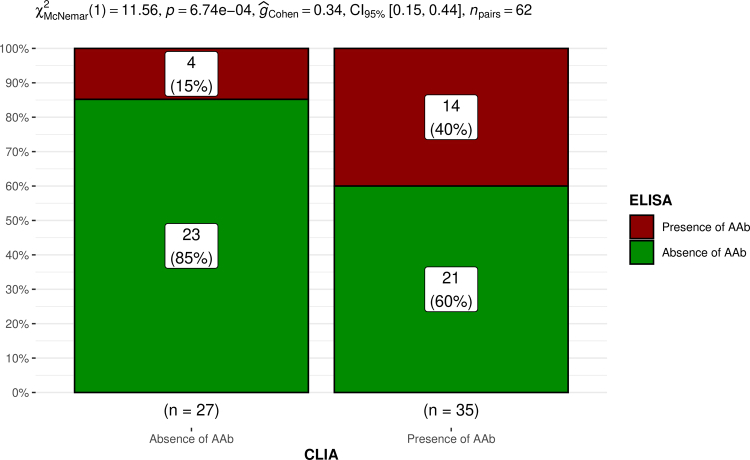


The analysis revealed significant disparity between the tests (*P* < .001), and the effect size was large (Cohen’s *g* = 0.34), as depicted in Supplementary Figure S4.

Upon segregating the analysis by clinical status, notable differences were observed primarily in samples obtained during remission. Figure 2 (left panels) illustrates that there was no significant discrepancy between tests during the acute phase (*P* = .08), whereas during the remission phase, the disparity was significant (*P* = .003) and large (Cohen’s *g* = 0.32).

Consequently, we excluded the acute phase and categorized the remission phase based on ADAMTS-13 activity (≥50% vs between 10% and 50%, Figure 2 [right panels]). In this categorization, the discrepancy was predominantly observed in the group with activity of <50%: in the group with activity of ≥50%, both tests consistently yielded negative results, while tests conducted in the group with activity of <50% exhibited a substantial and statistically significant difference once again.

Results from the evaluation of intertest agreement showed a moderate agreement in acute samples (Cohen’s *k* = 0.63) and no agreement in remission samples (*k* = −0.08). However, the absence of agreement was only in remission samples with an ADAMTS-13 activity of <50%, while remission samples with ADAMTS-13 activity of ≥50% showed a perfect agreement between the tests (Table).

**Table:** Intertest agreement rates between CLIA and ELISA AAb assays across various clinical conditions and ADAMTS-13 activity.

Clinical	ADAMTS-13 activity	Agreement (%)	Cohen’s *k*	Agreement (interpretation)
All	All	59.7	0.24	Slight
Acute	<10%	84.2	0.63	Moderate
Remission	≥10%	48.8	−0.08	None
Remission	>10% and <50%	33.3	−0.18	None
Remission	≥50%	100.0	ND	Perfect

AAb, anti–ADAMTS-13 autoantibody; CLIA, chemiluminescence immunoassay; ELISA, enzyme-linked immunosorbent assay.

### 3.3 HMWMs and von Willebrand activity

Patients in clinical remission were being monitored for their recovery and ongoing health status. The assessment included comprehensive ADAMTS-13 testing, as well as a detailed analysis of the HMWMs of VWF and their corresponding activity levels. The analysis of samples involved both qualitative and quantitative approaches.

Qualitatively, the multimer patterns were visually inspected to assess their distribution and structure. Quantitatively, densitometry analysis was used to measure the intensity of the bands representing the multimers. This provided a detailed assessment of the amount and distribution of each type of multimer (Supplementary Figure S5).

To establish normal reference intervals for HMWM, a study was conducted using 20 healthy volunteers. The primary aspect of this process involved comparing the intensity and distribution of the HMWM in their samples against those in a NPC used in each gel run.

No significant difference was observed in the mean percentage of AUC (%AUC) between controls and NPC, indicating that the levels of HMWM were within the normal range. These baseline data are crucial to provide a standard reference to identify abnormalities in patients with conditions like TTP.

Based on results of AAb, samples were categorized into 2 groups based on the concomitant activity level of ADAMTS-13 being <50% or ≥50% and compared with reference samples from healthy donors. Samples with ADAMTS-13 activity levels of ≥50% displayed normal multimer patterns upon visual inspection, unlike samples <50%.

Quantitatively (Figure 3A), samples with ADAMTS-13 activities of <50% and ≥50% and healthy donors had median HMWM %AUCs of 63.00%, 54.10%, and 56.05%, respectively (Figure 3A), with significant (*P* < .001) and very large (epsilon-squared = 0.27) differences among groups. Pairwise comparison indicated that samples with ADAMTS-13 activity level of <50% exhibit a significant difference with respect to both healthy donors and patients with activity level of ≥50% (*P* = .002), with a very large effect size (epsilon-squared = 0.27); no difference was observed between healthy donors and samples with ADAMTS-13 activity of ≥50%.

We also tested the samples for VWF activity, and the pattern of results was similar (Figure 3B: samples with ADAMTS-13 activities of <50% and ≥50% and healthy donors had mean VWF activities of 132.27%, 97.75%, and 94.82%, respectively). There were significant (*P* = .03) and large (omega-squared = 0.18) differences among groups. Pairwise comparison indicated that samples with an ADAMTS-13 activity of <50% differed significantly from samples with activity of ≥50% (*P* = .05) and healthy donors (*P* = .02).

### 3.4 Time series of patients with multiple relapses

In previous analysis, we observed that CLIA test for AAbs was often positive when ELISA was negative, especially in follow-up patients in remission with ADAMTS-13 activity between 10% and 50%. We explored whether such a positive CLIA test could anticipate the positive ELISA test and acute exacerbation. To do so, we identified 3 patients who had experienced multiple relapses, systematically collected from our biobank all available samples over a period of 3 to 5 years, and subjected them to CLIA testing for AAbs, comparing the results with ADAMTS-13 activity and AAb levels measured with ELISA.

Among these patients, a discernible association between the decline in ADAMTS-13 activity of <50% and the presence of AAbs measured by CLIA test was observed (Figure 4). Conversely, the ELISA test remained negative until the ADAMTS-13 activity was further diminished and sometimes even failed to detect any AAb.

The correlation requires further confirmation, but it could be significant in understanding the progression or exacerbation of iTTP. A reduction in ADAMTS-13 activity, along with functional evidence of autoantibodies, may indicate a deterioration of the condition and progression toward severe ADAMTS-13 deficiency. In cases where the autoantibodies are not present in sufiicient concentration to be detected by immunologic ELISA tests, but are already producing a biological effect, they can be detected by the functional CLIA test. Therefore, this finding supports the idea that the presence of ADAMTS-13 autoantibodies during remission predicts the risk of recurrence.

## 4 Discussion

The diagnosis of TTP requires a comprehensive evaluation that encompasses both clinical and laboratory aspects, with a focus on identifying specific markers and abnormalities associated with the disorder. This approach is essential for accurate diagnosis and timely initiation of appropriate treatment.

Clinical practice guidelines for the diagnosis and treatment of TTP have highlighted that the measurement of ADAMTS-13 activity and detection of autoantibodies are pivotal in confirming the diagnosis and distinguishing it from other conditions with similar clinical presentations [4]. Additionally, the International Working Group for TTP has developed updated consensus outcome definitions distinguishing between clinical remission and relapse. These are primarily defined by platelet count, while ADAMTS-13 remission and relapse are defined by ADAMTS-13 activity [23].

Consequently, there is an increasing demand for reliable and accurate tests that are accessible even in less specialized hospitals. Furthermore, clinicians need prompt results to tailor treatment in real-life practice.

The choice of method for assessing ADAMTS-13 may depend on factors such as laboratory resources, the availability of specific reagents, cost/labor efiiciency, and the patient’s clinical history. Clinicians and laboratory professionals select the most appropriate method based on these considerations to ensure accurate diagnosis and management of TTP.

Some potential advancements or changes in diagnosis that should be considered include improvements in the turnaround time, accuracy, and availability of ADAMTS-13 testing methods, making them more accessible and efiicient.

In this study, we assessed available and well-developed tests, not limited to specialized research laboratories, to evaluate our iTTP patients at diagnosis, during follow-up and clinical relapses.

In the preliminary comparison between ELISA and CLIA in ADAMTS-13 activity, the results confirmed the well-known almost perfect correlation [[7], [8], [9]]. Recent data from the External Quality Assessment Scheme (External Quality Control of Diagnostic Assays and Tests) for ADAMTS-13 activity testing also confirm results by reporting participant data with relatively low variability compared with those of other methods, including ELISA and fluorescence resonance energy transfer–based activity assays [24].

Compared with the analyses of activity assays, there has been instead a relatively limited reporting on the utilization of commercially available activity assays in conjunction with mixing studies (Bethesda assay) for the detection and quantification of AAbs [25].

ELISA is capable of detecting all AAbs regardless of their neutralizing or nonneutralizing properties and was compared with the functional Bethesda assay, where ADAMTS-13 activity is assessed through a functional test. There, the remaining activity of the test sample is determined as a percentage of that of the control sample exposed to identical incubation conditions. Our data show that the direct comparison of these methods revealed only a slight agreement, but the grouping of our patients based on their clinical condition and ADAMTS-13 activity levels showed distinct results ranging from moderate agreement in the acute patients to perfect agreement in clinical remission patients with ADAMTS-13 activity of ≥50%, suggesting that the tests are more interchangeable when the AAbs are present in high concentration or likely absent. However, it should be noted that in patients with a positive clinical prediction score for acute TTP and a negative functional AAb test, supplementation with ELISA assay is required to demonstrate the presence of noninhibitory autoantibodies [26]. Conversely, it is necessary to consider possible interference with Bethesda test by autoantibodies other than anti–ADAMTS-13 [27].

In the subgroup of patients in clinical remission with a decrease in ADAMTS-13 activity between 10% and 50%, we observed a notable difference in performance between the functional AAb test and immunologic assays for ADAMTS-13 autoantibodies. Specifically, the functional AAb test detected AAbs more frequently in this subgroup than the immunologic assay.

Additionally, in this subgroup of patients, the multimeric pattern of VWF exhibited a shift toward ultralarge VWF (ULVWF, a subgroup of HMWM). The multimeric pattern serves as a fingerprint during a highly dynamic process, indicating changes in the composition of VWF molecules, particularly an increase in larger multimers that represent the molecular basis of TTP. Indeed, it is crucial to always consider the levels or functions of VWF and ADAMTS-13 in relation to each other. It was previously described that a decrease in the ratio between ADAMTS-13 activity and VWF level can indicate a significant thrombotic risk. Therefore, a notable increase in VWF level alone, even without any decrease in ADAMTS-13 concentration, can still result in a considerable reduction of the ADAMTS-13/VWF ratio, potentially indicating or predicting a prothrombotic risk [3]. The predictive value of disease relapse determined by the presence of ADAMTS-13 AAb is still controversial, and in different studies, ADAMTS-13 AAb during remission emerged as one of the possible risk factors associated with an increased risk of iTTP relapse [28]. Previously, immune complexes have been identified in remission iTTP patients, which could potentially justify the higher frequency of positivity in the functional assay compared with the immunologic assay [29]. Indeed, it can be hypothesized that while ELISA captures the free autoantibodies, other forms of anti–ADAMTS-13 antibodies may exist in the form of immune complexes, which are not detected by the ELISA method but rather by the functional AAb test.

Additional evidence has been highlighted in some studies where it has been observed that autoantibodies can induce conformational changes in ADAMTS-13, where open ADAMTS-13 conformation in iTTP patients in remission is correlated with a decrease in ADAMTS activity. In summary, ADAMTS-13 conformation is open in the acute phase, while it is closed in remission if ADAMTS-13 activity is normal (>50 IU/dL); ADAMTS-13 antibodies purified from iTTP patients can induce the opening of ADAMTS-13 *in vitro* [30,31]. Therefore, a sustained open ADAMTS-13 conformation appears to be a novel biomarker of acute and subclinical iTTP and is closely associated with the presence of ADAMTS-13 antibodies, as well as with a decrease in the ADAMTS-13 activity.

A long-term follow-up of 3 patients with iTTP showed that decreases in ADAMTS-13 activity of <50% were preceded by a positive functional AAb test, which was followed later on by a clinical exacerbation of iTTP, and often by a positive immunologic test.

As previously demonstrated, the inhibition of ADAMTS-13 activity by IgG autoantibodies is the primary mechanism underlying severe deficiency of plasma ADAMTS-13 activity [32]. Our data underscore the strong connection between ADAMTS-13 antibodies (such as IgG trapped in ADAMTS-13/IgG immune complexes) that may be present and producing a biological effect *in vivo*, but undetectable *in vitro*. These cases may be connected to relapse, although the exact chronological cause/consequence process of this link remains unclear. Our study only presents anecdotal evidence to demonstrate that the functional AAb test of ADAMTS-13 can predict subclinical iTTP and the risk of relapse, and a dedicated larger clinical study is required. In summary, while consensus among published studies is lacking, evidence suggests that ADAMTS-13 autoantibodies could serve as markers for recurrence risk during the remission phase. Consequently, enhancing laboratory methods for identifying and characterizing these autoantibodies, alongside comprehensive data collection, will be pivotal for advancing our understanding of iTTP in the future.

## Figures and Tables

**Supplementary Figure S5:**