# Supplementary material for: A comparative study of anti–ADAMTS-13 antibody dynamics in immune-mediated thrombotic thrombocytopenic purpura
Source: Res Pract Thromb Haemost. 2024 Jul 22;8(5):102525. doi: 10.1016/j.rpth.2024.102525 (PMC11364000; doi:10.1016/j.rpth.2024.102525)
Supplement: Supplementary Data [file mmc1.docx]

Supplementary materials

Figure S1: Residual plot of the Passing-Bablok regression analysis. In x-axis the mean of estimated values of both methods, in y-axis, optimized residuals.

Figure S2: Comparison between ADAMTS13 activity as determined by CLIA or ELISA. Results from both frequentist and Bayesian statistical tests are annotated in the figure, respectively above, and below.

Figure S3: Comparison between the patients classified as positive vs negative in ADAMTS13 activity as determined by CLIA or ELISA. Results from statistical tests are annotated in the figure.

Figure S4: Comparison between the patients classified as positive vs negative in anti-ADAMTS13 Antibody as determined by CLIA or ELISA. Results from statistical tests are annotated in the figure.

Figure S5: Electrophoresis and densitometric analysis of von Willebrand factor multimers in pa- tients with iTTP in remission, classified according to their ADAMTS13 activity levels, above or below the 50% level. The densitometric analysis involved the assessment of low-molecular-weight multimers (LMWMs, peak 1-3), intermediate-molecular-weight multimers (IMWMs, Peak 4-7), and high-molecular-weight multimers (HMWMs, Peak >7). In comparison to the normal plasma control (NPC), both selected patients with ADAMTS13 levels below 50% exhibited a significant increase in HMWMs, considering the percentage of area under the curve (AUC%) in relation to the normal reference interval.
